# Pitch corrections occur in natural speech and are abnormal in patients with Alzheimer's disease

**DOI:** 10.3389/fnhum.2024.1424920

**Published:** 2024-08-21

**Authors:** Anantajit Subrahmanya, Kamalini G. Ranasinghe, Hardik Kothare, Inez Raharjo, Kwang S. Kim, John F. Houde, Srikantan S. Nagarajan

**Affiliations:** ^1^Department of Electrical and Computer Engineering, University of Illinois at Urbana-Champaign, Urbana, IL, United States; ^2^Department of Neurology, University of California, San Francisco, San Francisco, CA, United States; ^3^Department of Radiology and Biomedical Imaging, University of California, San Francisco, San Francisco, CA, United States; ^4^Department of Speech, Language, and Hearing Sciences, Purdue University, West Lafayette, IN, United States; ^5^Department of Otolaryngology—Head and Neck Surgery, University of California, San Francisco, San Francisco, CA, United States

**Keywords:** speech production, speech perception, speech control, auditory feedback, Alzheimer's disease

## Abstract

Past studies have explored formant centering, a corrective behavior of convergence over the duration of an utterance toward the formants of a putative target vowel. In this study, we establish the existence of a similar centering phenomenon for pitch in healthy elderly controls and examine how such corrective behavior is altered in Alzheimer's Disease (AD). We found the pitch centering response in healthy elderly was similar when correcting pitch errors below and above the target (median) pitch. In contrast, patients with AD showed an asymmetry with a larger correction for the pitch errors below the target phonation than above the target phonation. These findings indicate that pitch centering is a robust compensation behavior in human speech. Our findings also explore the potential impacts on pitch centering from neurodegenerative processes impacting speech in AD.

## 1 Introduction

Speakers unconsciously compensate for perturbations of pitch (Burnett et al., 1998), formant frequencies (Oschkinat and Hoole, 2020), and other acoustic cues (Raharjo et al., 2021) in their auditory feedback to maintain accurate speech production (Houde and Nagarajan, 2011, 2015). These previous studies have used real-time digital signal processing (DSP) programs to artificially perturb these various features of a subject's speech picked up by a microphone, which results in the perturbed auditory feedback in the participants' earphones. Subjects respond to these feedback perturbations by changing their speech output such that they compensate for the artificial auditory feedback perturbations.

Recent studies have shown that such online compensation may also occur in natural speech (i.e., speaking without any artificial perturbation): when a speaker makes repeated productions of a vowel, the formant frequencies at phonation onset vary somewhat across productions. If we compare the variance of the distribution of formant frequencies at phonation onset with the variance some time later in the utterance (say, for example, 100ms later), we find this variance is reduced. Thus, for each production of the vowel, initial deviance from the median is compensated for, reducing the deviance from the median as the utterance continues. This process is called “centering,” and has been shown to occur with formant frequencies (Niziolek et al., 2013). However, it remains unclear to what extent this correction applies to pitch and whether a comparable “pitch centering” mechanism exists.

In formant centering, a two-dimensional formant space created by the first two formants of a vowel is used to characterize the direction of deviations from the median as well as the degree of correction via centering. In contrast to this two-dimensional formant space, speakers perceive pitch as a one-dimensional scalar value (Larson et al., 2008). Nevertheless, it is reasonable to expect that the control of pitch could also show centering behavior during speaking.

If the pitch centering phenomenon exists, it would raise questions about whether and how it is affected in different populations. Alzheimer's Disease (AD) is a neurodegenerative condition that initially predominantly involves the fronto-temporal cortices, which are also key parts of the speech motor control network (Guenther and Hickok, 2015). As such, AD provides an excellent disease model to gain a deeper understanding of the mechanisms of speech motor control and the vulnerability of these mechanisms to the impairment of the frontal and temporal regions. A more immediate motivation for including AD in the current study is the result found by an earlier study that responses to pitch feedback perturbations–i.e., the pitch perturbation reflex - are impacted in patients with AD (Ranasinghe et al., 2017).

Here, motivated by previous research on feedback control abnormalities in pitch in AD (Ranasinghe et al., 2017), we demonstrate the existence of pitch centering, a speech feedback control mechanism, for the first time. We show that healthy elderly speakers and patients with AD exhibit pitch centering. Furthermore, by comparing the pitch production behavior of the elderly speakers with the age-matched AD patients, we also show how AD affects this centering behavior.

## 2 Methods

### 2.1 Participants

Thirteen patients meeting the diagnostic criteria for AD (McKhann et al., 2011) and 16 age-matched healthy volunteers participated in the study. The patients with AD in the current study are a subset of the 19 patients who participated in the altered feedback experiment reported in our previous work (Ranasinghe et al., 2019). The 16 control participants were the same set of subjects from our previous behavioral study. The participants were recruited from research cohorts at the University of California San Francisco (UCSF) Memory and Aging Center. All patients underwent a complete clinical evaluation, and the diagnosis was made at a multidisciplinary consensus meeting for each patient individually. To make our cohort more uniform and representative we excluded patients who fulfilled the current diagnostic criteria logopenic variant of primary progressive aphasia or posterior cortical atrophy syndrome (Mendez et al., 2002; Gorno-Tempini et al., 2011). Eligibility criteria for age-matched healthy participants included normal cognitive performance, normal structural brain imaging, and absence of neurological, psychiatric, and other major illnesses. Informed written consent was obtained from all participants or their assigned surrogate decision makers. The study was approved by the UCSF institutional review board for human research and the methods were carried out in accordance with the relevant guidelines and regulations.

### 2.2 Neuropsychological assessment

Both patients and controls underwent Mini-Mental State Examination (MMSE) (Folstein et al., 1975). In a structured caregiver interview, the Clinical Dementia Rating (CDR) scale, and CDR Sum of Boxes (CDR-SOB) were documented for each patient (Morris, 1993, 1997). All patients with AD underwent a battery of neuropsychological tests designed to assess major domains of cognition, including executive, fluency, memory, and language functions. The full battery of tests was detailed in previous reports (Ranasinghe et al., 2017). Statistical differences in demographic characteristics and neuropsychological test performance between the patients and controls were examined using SAS (SAS 9.4, SAS Institute Inc.).

### 2.3 Hearing status

All participants self-reported normal hearing and were assessed clinically for any hearing loss. Each participant underwent a bilateral tone hearing test to verify the hearing status and to confirm the proper earphone placement during the experiment.

### 2.4 Apparatus and procedure

The experiment consisted of two successive sessions, in which participants were asked to phonate a vowel multiple times (74 trials). In each trial, the participant reclined in the supine position in a MEG scanner and phonated the vowel /a/ into a MEG-compatible optical microphone (Phone-Or Ltd., Or-Yehuda, Israel) while listening to the real-time audio feedback via MEG-compatible earplug earphones (model ER-3A, Etymotic Research, Inc., Elk Grove Village, IL). This process incurred a feedback delay of 19 ms (Kim et al., 2023). During the trial, the pitch of the auditory feedback was perturbed for 400 ms following a randomly jittered delay of 200 - 500 ms from the phonation onset. An existing publication analyzes audio samples collected during and after the application of perturbation, while this paper's analysis interval includes only the first 200 ms after the phonation onset before any perturbations are applied.

Each trial began with the presentation of a clearly visible dot on a screen directly in front of the participant, serving as a visual cue for participants to produce the vowel /a/. Before the start of the experiment, the volume of auditory input through the earphones was adjusted to a comfortable level so that participants reported that their auditory feedback was heard at approximately the same loudness as what they would normally hear when speaking without wearing the earphones. This was to ensure that the participants perceived the auditory feedback through their headphones as natural. The participants produced the vowel sound for the duration of the visual cue displayed on the screen (2.5 s) and then stopped phonation for the next 2.5 seconds during which time the screen was blank. After every 15 trials participants were provided with an optional break time.

### 2.5 Data processing and analysis

#### 2.5.1 Acoustic data analysis

We used an autocorrelation-based pitch tracking method (Parsons, 1987) to extract a pitch-time-course measured in Hz from the raw acoustic recordings. Trials with pitch tracking errors or incomplete utterances were excluded (50.7% and 14.8% of recorded trials for patients with AD and controls, respectively). In both patients and controls, of the trials that were excluded, around 50% were excluded due to incomplete utterances, and the other 50% were excluded due to pitch tracking errors. Phonation onset for all trials was aligned to 0ms, and an analysis interval of 200ms was extracted.

**Figure 1:**
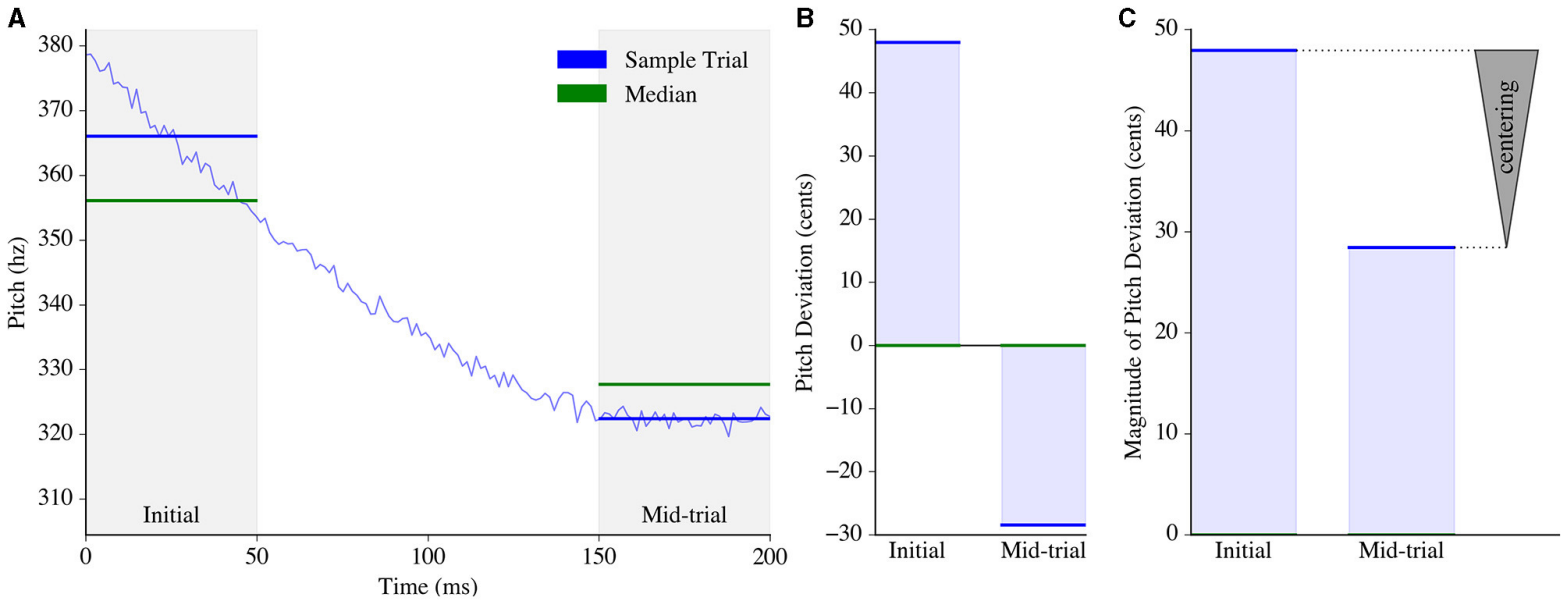
Pitch centering estimation process. **(A)** Pitch was tracked from 0 to 200ms for an example trial (blue). The average pitch, represented by horizontal blue lines, was then computed within two analysis windows: the initial window and the mid-trial window. For each window, the median of the averaged pitch measurements across all trials of that subject was calculated (green). **(B)** The average pitch for each window (in Hz) was then converted to pitch deviations from the median (in cents) using Equation 1 (*F*_trial, Hz_ and *F*_median, Hz_ represent the average trial pitch and across-trial median pitch for that window, respectively). **(C)** The magnitude of the pitch deviation was computed for each window. The measurement of “centering” in the trial was obtained by subtracting the magnitude of the mid-trial pitch deviation from the magnitude of the initial pitch deviation, depicted by an arrowhead with the base at the initial pitch deviation and the apex located at the mid-trial pitch deviation.

We defined the time window from 0ms to 50ms as the “Initial” window and the time window from 150 ms to 200 ms as the “Mid-trial” window (Figure 1A). For every trial, the average pitch in Hz within each time window was computed. These averaged pitch values were then normalized per subject by converting from hertz to cents to minimize the effect of absolute pitch.


(1) $$\begin{array}{l} F_{\text{trial,cents}}=1200\log_{2}\left(\frac{F_{\text{trial, Hz}}}{F_{\text{median, Hz}}}\right) \end{array}$$

Equation 1 describes the conversion of a pitch value from units of hertz *F*_trial, Hz_ to cents *F*_trial, cents_, relative to a given median pitch *F*_median, Hz_ computed across all trials of the subject for that particular window (Figure 1B). All subsequent mentions of initial and mid-trial pitch in this paper pertain exclusively to the pitch deviation from the median, as computed in cents.

Centering was then computed for each trial using Equation 2, where *F*_initial_ is the pitch (in cents) corresponding to the initial time window of that trial, and *F*_mid_ is the pitch (in cents) corresponding to the mid-trial time window. Figure 1C demonstrates how centering would be computed for an example trial given a predefined subject-wise median for each time window.


(2) $$\begin{array}{l} \text{Centering}=|F_{\text{mid}}|-|F_{\text{initial}}| \end{array}$$

Each trial was then classified as either “Upper Peripheral,” “Central” or “Lower Peripheral” based on the tercile of the trial's initial pitch relative to the subject's initial pitch distribution; trials with an initial pitch in the 0-33.3 percentile were Lower Peripheral, 33.4-66.5 were Central, 66.6-100 were Upper Peripheral.

#### 2.5.2 Statistical analyses

Trials with initial pitch values exceeding two standard deviations of the mean initial pitch were excluded from all statistical analyses. We first used the ks.test function from the stats package as implemented in R (version 4.2.2) (R Core Team, 2021) to run a Kolmogorov-Smirnov test of normality to confirm that the initial and mid-trial pitch distributions of patients and controls were normally distributed (<0.0001 in all cases). We then conducted a two-sided F-test in R comparing the initial and mid-trial pitch distributions for patients with AD and controls to analyze the impact of centering on pitch variability over time.

We analyzed the centering response for each trial, focusing on the impact of initial pitch (above vs. below the median). However, a direct comparison of the centering response across all three terciles could be misleading, as the central tercile inherently has lower-magnitude initial pitch values than the peripheral terciles for each subject. This discrepancy could result in significantly different magnitudes of perceived errors. To address this, we employed a linear mixed effects model using the lme function from R's nlme package with a compound symmetry correlation structure (Pinheiro and Bates, 2000). This model evaluated the interaction between group and tercile (peripheral or central), including subject identification as a repeated measure. Mixed models are robust for analyzing data with variable numbers of observations, as they account for both within- and between-subject factors, thereby providing a more accurate estimate of error. To account for trial-by-trial variability within participants and the variable number of analyzable trials between participants, we pooled all trials across subjects in each group (total number of trials: AD = 617; controls = 1,317) while maintaining subject identity.

We separately compared the centering response across trials and analyzed the effect of group and tercile on centering (this time where Tercile was either “Upper Peripheral” or “Lower Peripheral”) using a similar mixed model.

## 3 Results

### 3.1 Participant characteristics

Patients with AD were mild to moderately impaired with a mean CDR of 0.84 ± 0.24 (n = 4, CDR of 0.5; n = 9, CDR of 1) and a mean MMSE of 22.54 ± 3.62. Control participants were matched with AD patients in age, sex, handedness, race, and education (Table 1).

**Table 1:** Participant demographics.

	**Controls**	**AD patients**	***p*-value**
Age	64.00 ± 5.25	59.62 ± 7.59	0.0779
Female sex	11 (68.75%)	8 (61.54%)	0.7141
White race	15 (100.00%)	12 (92.31%)	0.4643
Education	18.0 (17.0–18.25)	18.0 (14.0-18.0)	0.2979
Right handedness	16 (100.00%)	10 (76.92%)	0.0783
CDR	0.0 (0.0–0.0)	1.0 (0.5-1.0)	<0.0001
CDRSOB	0.0 (0.0–0.0)	4.5 (4.0-5.0)	<0.0001
MMSE	30.0 (29.75–30.0)	23.0 (22.0-24.0)	<0.0001

Abbreviations: AD, Alzheimer's disease; CDR, Clinical Dementia Rating; CDR-SOB, CDR Sum of Boxes; MMSE, Mini-Mental State examination.

The AD cohort included four patients with CDR = 0.5 and nine patients with CDR = 1; Values for age are mean ± SD.

Age ranges are 49.36–77.45 and 56.22–75.56, for Alzheimer's disease patients and control participants respectively. Statistical tests were unpaired t-tests for age, education, CDR, CDR-SOB, MMSE; Fisher Exact test was used for sex, race, and handedness. Race was self-reported; one control participant and 1 patient with AD withheld from reporting race. Scores on the MMSE range from 0 to 30, with higher scores denoting better cognitive function.

### 3.2 Pitch centering is observed in healthy elderly and in patients with AD

**Figure 2:**
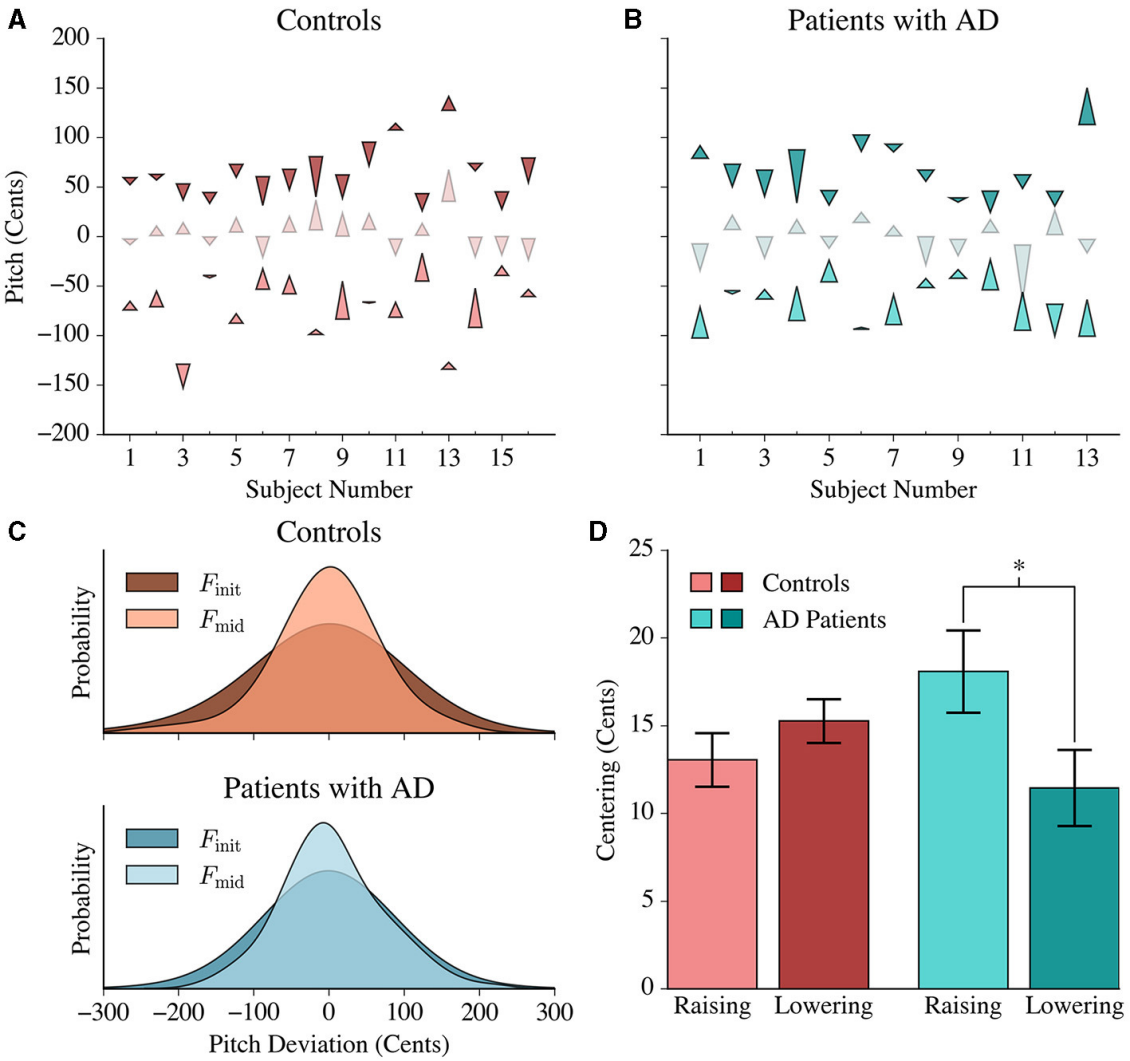
Pitch centering responses in controls and patients with AD. **(A)** Each control's average centering response is represented by three arrowheads. The lower, middle and upper arrows denote the average lower, middle and upper peripheral tercile responses, respectively. The base of each arrowhead corresponds to the average *F*_init_ value and the point of the arrowhead corresponds to the average *F*_mid_ value for each respective tercile. **(B)** The average centering response of each patient is shown using similar arrowheads to **(A)**. **(C)** Gaussian distributions were fit to the initial and mid-trial pitch values of both populations. Controls and patients with AD exhibited a greater variance in the initial pitch distribution compared to the mid-trial pitch distribution. This reduction in variance was more pronounced in the patient population than in controls. **(D)** Patients exhibited significantly greater centering for lower peripheral responses (raising their pitch) in comparison to the upper peripheral responses (lowering their pitch), while controls had a symmetric average response. The height of each bar represents the average centering response in the lower and upper peripheral trials, and the error bars correspond to the standard error within each tercile.

Both patients with AD and healthy participants exhibited pitch centering behavior. We quantified centering as the difference in magnitude of pitch deviation from 0 cents between the initial and mid-trial utterance for each trial (Figures 2A, B). Furthermore, the mid-trial pitch showed reduced variability compared to the initial pitch in both healthy controls and patients with AD, similar to patterns observed in past formant centering studies (Figure 2C; Controls: F_(1316, 1316)_ = 1.234, *p* < 0.001; AD: F_(616, 616)_ = 1.2486, *p* = 0.006) (Niziolek et al., 2013).

Based on the initial pitch in each trial per subject, we could identify the distribution of “lower peripheral” trials (where the initial pitch falls within the bottom tercile of the distribution), “upper peripheral” trials (where the initial pitch falls within the upper tercile of the distribution) and “central” trials, which are in-between. Figures 2A, B present the average pitch movement by tercile for every subject, represented by an arrow starting from the average initial pitch (*F*_init_) value to the average mid-trial pitch (*F*_mid_) value by tercile. The peripheral trials showed a larger centering response magnitude compared to the central trials [F_(1, 1903)_ = 566.385; *p* < 0.0001]. This is an expected phenomenon because more correction would be necessary for the upper tercile and lower tercile trials given that they have greater magnitude deviations from the median.

### 3.3 Patients with AD displayed asymmetric centering by raising vs. lowering pitch

In lower peripheral trials, positive centering would result in raising the pitch (“raising”) while in upper peripheral trials, positive centering would result in lowering (“lowering”) the pitch. The average centering response of controls was similar in both the lower (13.050±1.531) and upper terciles (15.267±1.244), while the patient group exhibited larger centering responses when raising pitch (18.083±2.33 cents) compared to when lowering pitch (11.454±2.16 cents) (Figure 2D). The results from the linear mixed model confirmed this interaction [F_(1, 1903)_ = 6.265, *p* = 0.012] between the upper and lower terciles for patients with AD.

**Figure 3:**
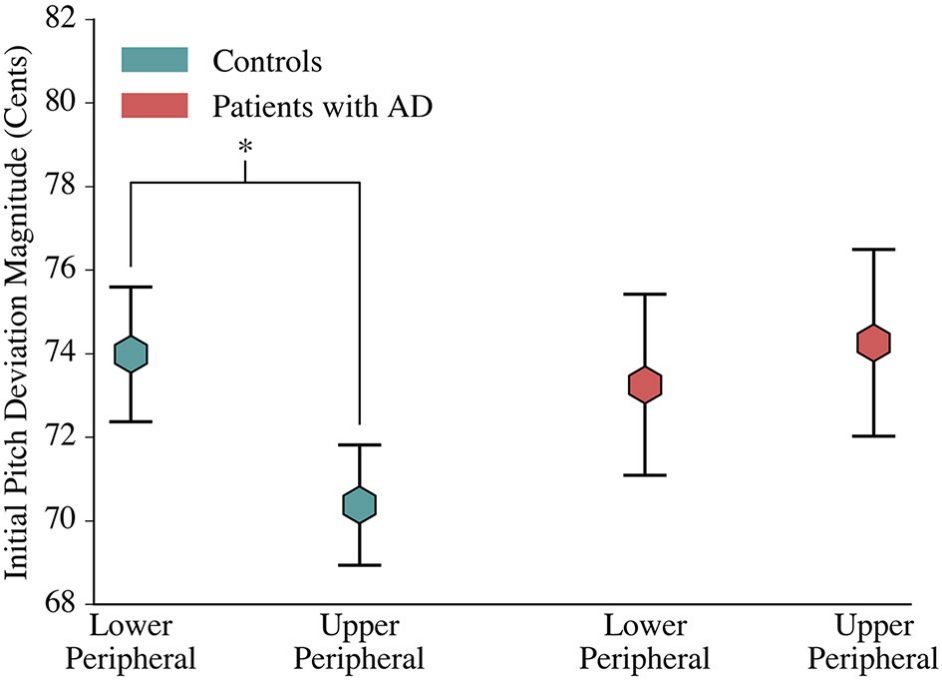
Comparison of Control and Patient Initial Deviations. At phonation onset, controls deviated further from the median production in lower peripheral trials than in upper peripheral trials. Patients with AD, however, had a symmetric initial pitch magnitude. The mean magnitude of initial pitch is depicted by hexagons, and error bars visualize the standard error.

One interesting finding was that the distribution of initial pitch errors was asymmetrical for controls and symmetrical for patients. Controls had statistically significant [F_(1, 1903)_ = 2.050, *p* = 0.048] greater magnitude lower peripheral errors (73.986 ± 1.612 cents) than upper peripheral errors (70.378±1.439 cents), yet had a symmetrical centering response. In contrast, patients had similar mean error magnitudes for the upper (73.253±2.168 cents) and lower (74.259±2.233 cents) peripheral terciles, yet had an asymmetrical centering response (Figure 3).

## 4 Discussion

Pitch centering is observed as the convergence of peripheral utterances to match a target (median) response. Both patients with AD and controls demonstrated this centering behavior in a manner consistent with recent findings in formant centering: trials that started far from the median moved inwards over time (Niziolek et al., 2013). Although controls had similar centering responses for trials originating in either of the peripheral terciles, the patient population exhibited a stronger centering effect in lower peripheral trials than in upper peripheral trials. This asymmetry cannot be attributed to differences in the initial pitch deviation from the median: patients had similar distributions for pitch errors above and below the median production while controls had an asymmetrical initial error distribution, where initial pitch deviations in lower peripheral trials were of greater magnitude than the deviations in upper peripheral trials.

### 4.1 A model of phonation control that explains initial pitch deviations and centering

In past work, a State Feedback Control (SFC) model was used to describe how the CNS controls phonation, specifically regarding the control of pitch (Houde and Nagarajan, 2011; Houde et al., 2014). The model gives a possible explanation for why there are errors in achieving an initial pitch target and describes mechanisms for the centering response that correct these initial errors. The model has a simple, one-dimensional “larynx” (modeling muscles controlling vocal fold length, e.g. the cricothyroid muscle) whose pitch can be changed by a phonation control network in the CNS (Houde et al., 2014). Within this network, the ventral premotor cortex (vPMC) maintains a running estimate of the current laryngeal state, which in this simple model consists of the current pitch and pitch velocity. From an efference copy of the current laryngeal controls, vPMC predicts the next laryngeal state, which in turn predicts the sensory feedback (both somatosensory and auditory) expected from the larynx. If actual sensory feedback does not match predictions, these feedback prediction errors are used to correct the predicted state, resulting in an updated estimated laryngeal state that is fed back to M1. M1 compares the pitch of the estimated laryngeal state with the desired pitch specified by the higher frontal cortex and issues laryngeal controls that specify the desired change in pitch produced by the larynx.

These desired pitch changes specified by laryngeal controls descending from the cortex are integrated by the lower motor system of the larynx into the current pitch output. However, these descending cortical controls are also first somewhat corrupted by signal-dependent noise (i.e., noise that scales with the size of the cortical control signals) before being integrated into the current pitch output. In the model of the larynx, no pitch is represented by a pitch value of zero. Thus, at phonation onset, the desired pitch change specified in the descending cortical controls is large: e.g. a change from 0 (no pitch) to 120 Hz, for a male voice. As a result, the signal-dependent noise added to the desired pitch change is also large, and integrating this noise-corrupted desired pitch change results in an output pitch at phonation onset that deviates from the target pitch. Because the signal-dependent noise processes are thought to arise at the very lowest levels of the motor system (e.g., as noise in the number of motor units recruited to implement the desired pitch change), they are not anticipated by the state prediction process in vPMC. As a result, sensory feedback of the initial pitch output mismatches the sensory feedback predictions derived from the state prediction, and the resulting feedback prediction errors (both somatosensory and auditory) generate corrections to the predicted laryngeal state that, when fed back to M1, cause it to output laryngeal controls that are pitch changes to correct for the initial pitch errors. Note also that as these corrective pitch changes are much smaller than the initial pitch change, they are corrupted with correspondingly much smaller signal-dependent noise, meaning that this correction process is a convergent process, and is what we call centering.

### 4.2 Asymmetric centering behavior could arise from either perceptual sensitivity or motor effort asymmetry in AD patients

One potential explanation for the asymmetrical centering response of AD patients in peripheral trials is that the AD patient population may be more sensitive to pitch deviations below the median than above the median. In turn, patients would be more likely to attempt to correct pitch errors in lower peripheral trials than those in upper peripheral trials of similar magnitude, leading to increased mean centering responses for the former.

Alternatively, raising pitch may require more muscular effort than lowering pitch. Agonist-antagonist pitch production models separate physiological sources of pitch change, allowing for quantitative changes in motor unit recruitment to provide a notion of increased/decreased effort to realize a particular change in pitch (Gerazov and Garner, 2016). Patients with AD may require more effort to lower their pitch than raise their pitch for similar deviations from the target pitch value. Assuming the pitch control system factors in muscular effort when regulating pitch, this asymmetry in muscular effort would translate to a reduction in centering for upper peripheral trials and enhanced centering for lower peripheral trials.

### 4.3 Limitations and future directions

Since our study did not isolate the various feedback sources, we cannot determine whether the contrasting AD and control results stemmed from differences in sensory feedback sensitivity, asymmetric muscular effort required for pitch changes, or a combination of both. Future functional neuroimaging analysis could help quantify the motor unit recruitment changes and provide more conclusive evidence for the mechanisms responsible for the centering irregularities in patient populations.

Although this small study had limited age and racial diversity among participants, it is an important step toward establishing pitch centering behavior and characteristics, improving our understanding of speech motor control. Additional studies observing similar correlations in non-Caucasian and younger subjects would help further validate the characteristics of centering in controls and patients with AD for a more general population.

## Data Availability

The data analyzed in this study is subject to the following licenses/restrictions: Anonymized subject data will be shared on request from qualified investigators for the purposes of replicating procedures and results, and for other non-commercial research purposes within the limits of participants' consent. Correspondence and material requests should be addressed to the corresponding author. Requests to access these datasets should be directed to srikantan.nagarajan@ucsf.edu.
